# The Longitudinal Relationship Between Physical Functions and Cognitive Functions Among Middle-Aged and Older Adults in Primary Care

**DOI:** 10.3390/ijerph22060908

**Published:** 2025-06-06

**Authors:** Nan Hu, Wupeng Yin, Rabeya Illyas Noon, Noof Alabdullatif

**Affiliations:** 1 Department of Biostatistics, Robert Stempel College of Public Health and Social Work, Florida International University, Miami, FL 33199, USA; wyin@fiu.edu (W.Y.); rnoon@fiu.edu (R.I.N.); 2 Department of Family and Preventive Medicine, University of Utah School of Medicine, Salt Lake City, UT 84132, USA; 3 Department of Global Health, Robert Stempel College of Public Health and Social Work, Florida International University, Miami, FL 33199, USA; nalab008@fiu.edu

**Keywords:** aging, middle-aged and older adults, cognitive decline, cognitive functions, handgrip strength, gait speed, longitudinal study

## Abstract

(1) Background: Gait speed (GS) and handgrip strength (HGS) have been identified as factors in cognitive impairment, depression, sleep problems, and quality of life. This study aims to comprehensively investigate the longitudinal relationship between physical functions (gait speed and handgrip strength) and cognitive functions, as well as cognitive decline, in middle-aged and older adults in China. (2) Methods: Using longitudinal data from the China Health and Retirement Longitudinal Study (CHARLS), we analyzed 1903 subjects aged 60 and above with repeated measurements of GS, and 4218 subjects aged 45 and above with repeated measurements of HGS. The cognitive functions we considered included drawing ability, word recall, TICS-10, and an overall cognitive score (OCS). Mixed-effect logistic and linear regression were used to analyze the association between GS/HGS and cognitive functions in middle-aged and older adults with repeated measurements. (3) Results: A faster GS is associated with better drawing ability (OR = 1.33, *p* = 0.045) and TICS-10 (OR = 1.60, *p* = 0.024). A stronger HGS is associated with higher odds of drawing ability (OR = 1.36, *p* = 0.012) and better TICS-10 (OR = 1.41, *p* = 0.018). Both weak HGS and slow GS are significantly associated with a higher decline in cognitive function, while HGS is more predictive of the decline for women and GS is more predictive for men. (4) Conclusions: Both GS and HG are positively associated with cognitive functions cross-sectionally and longitudinally in middle-aged and older adults. Health interventions targeting HGS and GS may help improve cognitive functions. Future research is warranted to establish the causal relationship between these interventions and improvements in cognitive functions.

## 1. Introduction

The global population’s increasing life expectancy and population aging pose significant challenges to society and the economy, particularly in the public health system, causing health issues like cognitive decline, depression, and multimorbidity [1,2,3]. Thus, addressing various cognitive impairment risk factors is crucial for determining prevention methods that minimize their adverse effects.

Handgrip strength (HGS), a common measure for assessing overall muscular strength, has been identified as an early indicator of cognitive decline [4,5]. Recent studies have demonstrated that higher HGS in middle-aged and older adults is associated with better cognitive performance over time [6]. Similarly, lower HGS has been linked to an increased risk of cognitive impairment among older adults [7]. In addition, asymmetry in HGS, or a significant difference in strength between dominant and non-dominant hands, has been found to be associated with a higher risk of cognitive impairment [8]. It has been suggested that HGS measurements might be useful for early risk stratification and broader assessment of cognitive function [9,10].

Among middle-aged and older adults, the literature has reported that HGS can explain or is associated with overall strength, upper limb function, bone mineral density, fractures, falls, malnutrition, cognitive impairment, depression, sleep problems, diabetes, multimorbidity, and quality of life [11]. Research findings also indicate that stronger HGS in older adults is significantly associated with better cognitive function performance, as reflected by global cognitive scores and in various domains such as memory, language, attention, and cognitive decline. Yang and colleagues [12] evaluated the association between HGS and cognitive function in cancer survivors aged over 60 using two-wave data from the US National Health and Nutrition Examination Survey (NHANES) from 2011 to 2014 and found that grip strength was associated with many aspects of cognitive function among the cancer survivors. A recent study [13], using the Korea Longitudinal Study of Aging Panel Data, concluded that HGS is one of the most significant factors for predicting the progression to dementia, based on a nomogram for reversion to normal cognition. Prokopidis et al. [14] evaluated the association between HGS and learning and verbal fluency in older men without dementia through a cross-sectional study using the NHANES data from 2011 to 2014. The investigators concluded that higher HGS was independently associated with better learning ability for novel verbal information and verbal fluency among US men over the age of 60 and without dementia. The authors also suggested that longitudinal studies are needed to confirm whether muscle strength predicts cognitive function changes in older adults in a sex-specific manner, and whether this connection may be attributed to reverse causation due to declines in physical activity levels in the preclinical phase of dementia.

Gait speed (GS) is also recognized as a marker of higher-order cognitive function, including motor abilities, attention, and memory [15,16]. Walking GS is a dependable, noninvasive, and straightforward parameter that can be incorporated into routine elderly care as a part of comprehensive assessment for older adults [17,18]. Compared to traditional diagnostic methods like brain imaging and biological biomarkers, it is a more economical and quicker tool for standard dementia diagnosis [17,19]. Cognitive symptoms are frequently preceded by gait impairment, which may be an early indicator of cognitive decline [20,21]. Recent studies have established significant correlations between GS and global cognitive function and cognitive domains among older adults [22].

Additionally, variations in walking pace were found to be associated with loss in certain cognitive domains, including memory and executive function, reflecting the complex relationship between physical mobility and cognitive well-being [23]. Slower GS has been identified as a predictor of numerous risk factors, including dementia, emphasizing the importance of gait assessment in early detection strategies [23,24]. These findings emphasized the necessity of regularly monitoring GS and cognitive abilities in elderly individuals. Interventions designed to enhance physical mobility may positively impact cognitive health, thereby contributing to the overall well-being of the elderly population.

GS was identified as an important factor associated with aging-related cognitive functions among middle-aged and older adults. For example, using a 10-year follow-up dataset from the Japanese National Institute for Longevity Science–Longitudinal Study of Aging (NILS-LSA), Chou and colleagues [25] found that slow GS could predict 10-year cognitive decline, based on the Digit Symbol Substitution Test (DSST), and low HGS could predict 10-year cognitive decline, using the Mini-Mental State Examination (MMSE) as the metric. The authors claimed that GS and HGS are linked to cognitive decline, but there may be different mechanisms between brain and physical functions. In addition, a recent study by Gunnarsson et al. [26] found that cognitive test scores had a statistically significant association with three physical functions among 292 patients referred to an outpatient clinic for more than or equal to 3 months after acute COVID-19 infection. The authors found a weak to moderate association between physical and cognitive function.

Although the association between physical functions, such as HGS and GS, and cognitive functions has been extensively investigated, a relatively small proportion of the studies used longitudinal data with a large sample size, especially studies of middle-aged and older adults using primary health care. The association between physical and cognitive functions was established primarily based on cross-sectional data. Cross-sectional data make it impossible to examine the lagged effect and assess the impact of baseline physical functions (HGS and GS) on the cognitive function changes in middle-aged and older adults.

Our study aims to comprehensively investigate the longitudinal relationship between cognitive and physical functions (focusing on HGS and GS). We believe this study is a comprehensive investigation of their longitudinal relationship because this study not only examines the relationship between cognitive functions and concurrent HGS and GS, but also assesses the relationship between cognitive functions and previous HGS and GS. In addition, we investigate the association between temporal declines in cognitive functions and baseline physical functions, and perform the analysis stratified by gender. This study uses data from middle-aged and older adults (age 60 and above for GS, and age 45 and above for HGS) who participated in the China Health and Retirement Longitudinal Study (CHARLS) and self-reported as primary care users. The longitudinal study design allows us to investigate the relationship between cognitive functions and physical functions measured at the same time and at an earlier time (lagged effect) and facilitates the analysis of the impact of baseline physical functions on subsequent changes in cognitive functions. This study can shed light on how essential physical functions (i.e., HGS and GS) impact concurrent and future cognitive functions and predict future cognitive decline in middle-aged and older adults using primary care. It will also provide primary care providers (PCPs) and policy makers with an evaluation of HGS and GS as prediction tools for future cognitive impairment among middle-aged and older adults.

## 2. Materials and Methods

### 2.1. Data Source and Study Population

The CHARLS study is a nationally representative longitudinal study focusing on individuals aged 45 years and above residing in mainland China. The initial baseline survey was conducted in the period of 2011–2012, with subsequent follow-up waves in 2013 (Wave 2), 2015 (Wave 3), 2018 (Wave 4), and 2020 (Wave 5). The baseline survey covered 28 provinces, 150 counties/districts, and 450 villages/urban communities, ensuring national and regional representation of China’s middle-aged and older populations [27]. CHARLS utilized a stratified, multi-stage sampling strategy with probability proportional to size (PPS), incorporating stratification by region, urban versus rural status, and GDP per capita. The baseline sample was weighted to align closely with the demographic characteristics from China’s 2010 Population Census, thereby confirming its national representativeness. Ethical approval for all survey waves of CHARLS was provided by the Institutional Review Board (IRB) at Peking University, with the main household survey granted approval number IRB00001052-11015.

Our longitudinal study extracts data from Waves 1, 2, and 3 of CHARLS. Wave 1 was designated as the baseline, while Waves 2 and 3 served as follow-up assessments, as these three waves contained GS data (N = 24,752). Wave 2 was used as the baseline for those with longitudinal measurements but with missing data for Wave 1.

**Figure 1:**
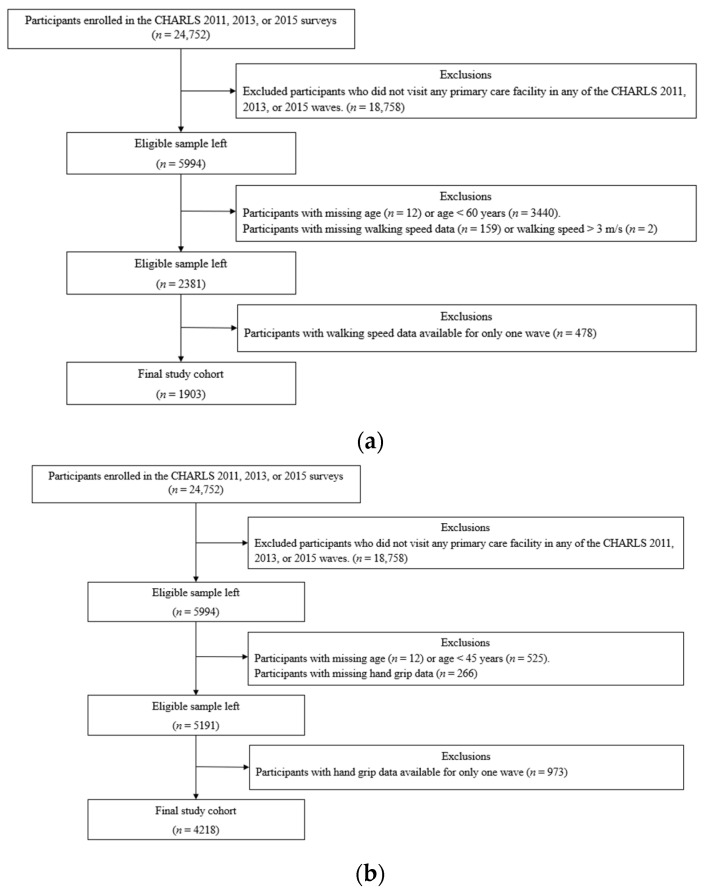
(**a**): Flowchart depicting the data selection and exclusion process for the GS cohort. (**b**): Flowchart depicting the data selection and exclusion process for the HGS cohort.

To establish the cohort for longitudinal measurements of GS, we specified the following inclusion criteria: (i) subjects using any primary care facility during the study period; (ii) subjects 60 years of age or older (only subjects 60 years of age or older have GS measurements in CHARLS); (iii) subjects with at least two measurements of GS among Waves 1, 2, and 3 of CHARLS. Exclusion criteria included the following: (i) subjects with a GS beyond the normal range (>3 m/s, or the 99th percentile of GS measurements in CHARLS Waves 1, 2, and 3); (ii) subjects without age information from any of the three waves. Among the initial 24,752 subjects, 18,758 individuals did not have access to any primary care facility during the study period; 3452 individuals did not have age information or were younger than 60 years of age; 161 individuals were missing GS measurements or had one or more recorded GS measurements exceeding 3 m/s; and 478 individuals had GS measurements available from only one of the three waves. The study cohort of GS comprises 1903 individuals, which is summarized in Table 1a. A detailed overview of the selection process is presented in Figure 1a.

To establish the cohort with longitudinal measurements of HGS, we used the following inclusion criteria: (i) subjects using any primary care facility during the study period; (ii) subjects 45 years of age or older; (iii) subjects with at least two measurements of HGS across Waves 1 to 3 of CHARLS. Exclusion criteria included the following: (i) subjects without age information from any of the three waves. Among the initial 24,752 subjects, 18,758 individuals did not have access to any primary care facility during the study period; 537 subjects did not have any age information or were younger than 45 years of age; 266 individuals missed HGS measurements for all three waves; and 973 individuals had HGS measurements available from only one of the three waves. The final study cohort of HGS consisted of 4218 subjects, which is summarized in Table 1b. A detailed overview of the selection process is presented in Figure 1b.

The GS cohort includes only older adults (aged 60 and above), while the HGS cohort includes both middle-aged and older adults (aged 45 years and above).

### 2.2. Measurements

#### 2.2.1. Measurement of Cognitive Function

Cognitive function was evaluated by using three measuring dimensions, which included visual-spatial ability, episodic memories, and mental status. These dimensions were quantified using the pentagon figure-drawing test, word recall test, and the Telephone Interview for Cognition Status (TICS-10) test, respectively [28,29,30].

Figure-drawing ability evaluation was based on the pentagon drawing test (PDT), which is widely employed in clinical and research environments to assess cognitive impairment as a component of the Mini-Mental State Examination (MMSE) [31]. PDT has been used to measure a person’s capacity to recognize visuospatial relationships among various items. The respondents were asked to reproduce a figure of two overlapping pentagons. The variable of drawing ability was coded as 1 for drawing the picture correctly, and 0 otherwise.

The word recall test is a measure of episodic memory. In CHARLS, both immediate and delayed recall tasks were assigned to study participants, who were asked to memorize 10 unique Chinese nouns drawn from one of four categorized lists. Respondents received a score based on the number of correct recalls. The final score, ranging from 0 to 10, was calculated as the average of immediate and delayed recall scores. In our analysis, the word recall variable was a binary outcome, dichotomizing the original word recall score (on a 0–10 scale) at the sample median. The binary outcome was coded as 1 for high and 0 for low word recall ability.

The TICS-10 score is based on the Telephone Interview for Cognitive Status (TICS), a general mental status assessment test. TICS has been utilized as a reliable and effective telephone screening tool for dementia and Alzheimer’s disease (AD) and shares many characteristics with the Mini-Mental State Examination (MMSE) [32,33]. The cognitive domains of TICS-10 in CHARLS include concentration, the orientation of time, and mathematical skills. There were 10 questions: today’s date, week, month, year, and season; subtract 7 from 100, and then sequentially subtract 7 from each result up to four times. The TICS-10 score was calculated as the total number of correct responses, ranging from 0 to 10. In our analysis, TICS-10 was dichotomized using the median score at baseline as the cutoff, with 1 indicating a high and 0 indicating a low TICS-10 score. The TICS can be administered over the telephone or conducted face-to-face [33]. In CHARLS, this test was conducted face-to-face with study participants in their homes.

The overall cognitive score (OCS) is the sum of the scores of the above three measurements. OCS is a comprehensive assessment of subjects’ cognitive statuses, and ranges from 0 to 21, with a higher score indicating better overall cognitive function [34,35,36]. In our study, OCS is used as a continuous-scale variable. In our exploratory analysis, OCS measurements are shown to be approximately normally distributed. Hence, we treated OCS as a normally distributed variable in data analyses.

#### 2.2.2. Assessment of GS

In CHARLS, walking GS tests were administered to respondents aged 60 and older. During these assessments, the time (in seconds) required to walk 2.5 m along a 4 m, non-carpeted walking course was recorded for both the first and second trials. GS was calculated by dividing 2.5 m by the shortest time recorded across the two trials. The median walking GS was used to categorize individuals into two groups: low GS (≤0.65 m/s) and high GS (>0.65 m/s).

#### 2.2.3. Assessment of HGS

In each wave of CHARLS, HGS for both hands were measured twice with Yuejian^TM^ WL-1000 dynamometer equipment (manufactured by Nantong Yuejian Physical Measurement Instrument Co., Ltd., Nantong, China) in kilograms [27]. Every participant received the same instructions for the handgrip test. The handgrip test was performed in a standing position with the elbow at a right angle, holding the dynamometer. The participants were asked to squeeze the dynamometer as hard as possible and hold it for a few seconds. The maximum of the four measurements was used in each wave for statistical analysis in this study [37,38]. To categorize participants by HGS, the median value was calculated based on the sample distribution. Participants were then classified into two groups as follows: low HGS (≤30 kg) and high HGS (>30 kg).

Beginning in Wave 4, CHARLS discontinued the collection of biomarker data, including all timed walking and strength measurements [27]. Hence, GS and HGS are available only in Waves 1 to 3. This explains why our study includes data only from the first three waves.

Physical function measurements, including GS and HGS, were conducted in respondents’ households by trained CHARLS county-level interviewers using standardized equipment and protocols. Interviewers followed detailed procedures outlined in the CHARLS 2011–2012 National Baseline Users’ Guide, including obtaining verbal confirmation from participants regarding safety and understanding before proceeding with each measurement [39].

#### 2.2.4. Covariates

The covariates that could confound the main effect were considered: the time-period effect (in years), baseline age (in years), gender (female = 1), education level (upper secondary education, vocational training, and tertiary = 1), BMI, and household income. BMI was classified into the following four categories according to the WHO recommendation for the Asian and South Asian populations [40]: underweight (≤18.5 kg/m^2^), normal weight (18.5 kg/m^2^ to 23.9 kg/m^2^), overweight (24 kg/m^2^ to 25 kg/m^2^), and obese (≥25 kg/m^2^). Household income was defined as the total income at the household level, including earnings, capital income, pension income, government transfers, other sources of income, and the combined income of all household members. The values are reported in Chinese Yuan (¥) in the unit of CNY 10,000. Household income was then dichotomized into low (≤0.56) and high (>0.56) categories using the median value from the baseline wave as the cutoff.

### 2.3. Statistical Analysis

The characteristics of the study population were summarized using frequencies (N) and percentages (%) for categorical variables and means ± standard deviations (SD) for continuous variables that were normally or approximately normally distributed. Chi-squared (χ^2^) tests and two-sample Student’s *t*-tests were employed to assess differences in covariates between HGS (high vs. low) status and between GS (high vs. low) status.

To enhance interpretability and ensure model stability, several key variables—including GS, HGS, and cognitive function measures—were dichotomized based on sample medians. This approach is commonly used in aging and epidemiological studies, particularly when clinical cutoffs are not standardized. Dichotomization also helped address issues of skewed distributions and limited variability in certain outcomes, such as drawing ability and TICS-10 scores, thereby improving model convergence and comparability across measures.

Given the hierarchical (multilevel sampling) structure of the CHARLS data, where individuals (Level 1) are repeatedly measured over time (within-subject correlation), nested within households (Level 2) and within communities (Level 3), generalized linear mixed-effects models (GLMMs) with random intercepts were employed. GLMMs are well-suited for analyzing multilevel longitudinal data, accounting for correlations both within individuals over time and across higher levels of clustering [41]. To properly model repeated measures within individuals, we incorporated a random intercept at the individual level, capturing within-subject correlation. Additionally, random effects at the household and community levels were included to adjust for contextual dependencies, ensuring more precise estimations of fixed effects [42,43]. Compared to traditional statistical models, GLMMs naturally accommodate the hierarchical and longitudinal data structure, making them ideal for examining associations between GS/HGS and cognitive function over time.

For categorical outcomes, the GLMMs estimate the odds ratios (ORs), 95% confidence intervals (CIs), and *p*-values of the fixed effects using maximum likelihood estimation with bound optimization by quadratic approximation. For the continuous outcome OCS, the LMM estimated the regression coefficients, 95% CIs, and *p*-values of the fixed effects. The regression model is presented in Equation (1):

(1) $$g\left(E\left(y_{ijkt}|u_{i},\;u_{j\left(i\right)},u_{k\left(j\left(i\right)\right)}\right)\right)=X_{ijkt}\beta +u_{i}+u_{j\left(i\right)}+u_{k(j(i))}+e_{ijkt}$$

where ${\;y}_{ijkt}$ represents the outcome for the kth individual in the jth household within the ith community, at time t;$\;X_{ijkt}$ is the design matrix of fixed effects, including exposure, time since baseline, and covariates being adjusted for; β is a vector of fixed-effect regression coefficients; $u_{k(j(i))}\;\sim \;N\left(0,\;\sigma_{in}^{2}\right)$ is the random part of the intercept at the individual level, capturing within-subject correlation due to repeated measurements;$\;u_{j}\;\sim \;N(0,\;\sigma_{h}^{2})$ is the random part of the intercept for households, accounting for shared household effects; $u_{i}\;\sim \;N(0,\;\sigma_{c}^{2})$ is the random part of the intercept for communities, controlling for broader contextual variation; and $e_{ijkt}\;\sim \;N(0,\;\sigma_{e}^{2})$ is the residual error. For binary outcomes, a logistic link function was used such that $g\left(x\right)=ln(\frac{x}{1-x})$.

To compare the temporal trend (slope) of the OCS between the HGS status groups (and GS status groups), we used a multilevel linear mixed-effects model that included the follow-up time (time since baseline in year), baseline HGS (or GS), and the interaction between follow-up and HGS (or GS). The interaction term would then be used to evaluate if the baseline HGS (or GS) could significantly impact the temporal trend of the cognitive score over time. The adjusting variables in the model are the same as those considered in model (1). Since the follow-up time is only over three CHARLS waves and our exploratory study did not find any non-linear trend in the OCS over this period, we do not consider the non-linear term (such as the quadratic or cubic term of the follow-up time) in the regression model.

All statistical tests were two-sided, and outputs with *p*-values < 0.05 were considered as statistically significant results. Data management and statistical analyses were performed using statistical package SAS (SAS Inst. Inc., Cary, NC, USA) version 9.4 and statistical package R (cran.r-project.org) version 4.4.2 SAS (SAS Inst. Inc., Cary, NC, USA) version 9.4.

### 2.4. Reporting

We followed the STrengthening the Reporting of OBservational studies in Epidemiology (STROBE) guidelines for cohort, case-control, and cross-sectional studies to report our study findings (https://www.strobe-statement.org, accessed on 29 April 2025).

## 3. Results

### 3.1. Sample Characteristics

A total of 1903 subjects were included in the study cohort for GS. The baseline characteristics of this cohort are summarized in Table 1a. The mean age of the study cohort was 67.43 ± 5.99 years. Slightly more than half of the study subjects were females (N = 1023, or 53.76%). Most of the study subjects had an education level less than lower secondary school (N = 1842, or 96.79%). About 44.82% of the subjects (N = 853) had normal weights. Individuals with high GS had statistically significant differences from those with low GS in all variables summarized in the table, except for BMI (*p* < 0.001 for all variables except for BMI, *p* = 0.499).

A total of 4218 subjects were included in the study cohort of HGS. The baseline characteristics of this cohort are summarized in Table 1b, and the mean age of the study cohort was 59.38 ± 9.22 years. More than half of the study subjects were female (N = 2462, or 58.37%). The majority of the study subjects had an education level less than lower secondary school. About 40% of the subjects (N = 1710) had normal weights. Individuals with high HGS had statistically significant differences from those with low HGS in all variables summarized in the table (*p* < 0.001 for all variables).

### 3.2. Association Between GS and Cognitive Functions

GS is statistically significantly associated with all cognitive outcomes measured concurrently (measured at the same timepoint as GS) in both unadjusted and adjusted models. Table 2a examines the associations between GS and cognitive outcomes measured concurrently within the same wave, reflecting the relationship between physical function and cognition at the same time point (concurrent effect). The result illustrates the OR, 95% CI, and *p*-values from the multilevel mixed-effects logistic regression model, which examines the association between GS and the odds of drawing ability, word recall ability, and TICS-10. Table 2a also reports the difference in mean, 95% CI of the difference, and *p*-values from the multilevel linear mixed-effects model for the association between GS and the continuous-scale OCS. Both univariable (unadjusted model) and multivariable analyses results are shown in Table 3a.

In the unadjusted model (Model 1), high GS is associated with 89% higher odds of drawing ability (OR = 1.89; 95% CI: 1.58 to 2.27; *p* < 0.001), 67% higher odds of word recall ability (OR = 1.67; 95% CI: 1.17 to 2.40; *p* = 0.005), and 131% higher odds of TICS-10 performance (OR = 2.31; 95% CI: 1.85 to 2.89; *p* < 0.001). For OCS, subjects with high GS, on average, have a 0.78 (difference in mean = 0.78; 95% CI: 0.57 to 0.99; *p* < 0.001) higher mean of the overall cognition score than those with low GS.

In the multivariable model adjusting for follow-up time, baseline age, gender, education level, BMI, and household income, high GS is associated with 33% higher odds of drawing ability (OR = 1.33; 95% CI: 1.07 to 1.66; *p* = 0.010), 119% higher odds of word recall ability (OR = 2.19; 95% CI: 1.14 to 4.18; *p* = 0.018), and 52% higher odds of TICS-10 performance (OR = 1.52; 95% CI: 1.15 to 2.00; *p* = 0.003). For OCS, subjects with high GS, on average, have a 0.57 (difference in mean = 0.57; 95% CI: 0.32 to 0.82; *p* < 0.001) higher mean of the OCS than those with low GS.

The results shown in Table 2b, in contrast, adopt a longitudinal perspective by examining whether physical function in a previous wave (e.g., GS at Wave 1) predicted cognitive performance in the subsequent waves (e.g., cognitive function at Waves 2 and 3). The result illustrates the OR (or mean difference), 95% CI, and *p*-values from the mixed-effects models that examine the association between the cognitive outcomes and previous GS (lagged by one wave). Estimates for both previous GS and current GS (as an adjusting variable) are reported in Table 2b. The previous (lagged) GS is statistically significantly associated with all cognitive outcomes measured concurrently, in the model that only adjusts for the current GS (Model A). In the multivariable model adjusting for current GS, follow-up time, baseline age, gender, education level, BMI, and household income (Model B), the lagged GS is not significantly associated with any of the cognitive functions. However, in these models, the current GS is significantly associated with all cognitive functions except for drawing ability in Model B.

### 3.3. Association Between HGS and Cognitive Functions

When only the concurrent HGS is considered in the model, HGS is statistically significantly associated with all cognitive outcomes in the unadjusted model and is significantly associated with all except for word recall in the adjusted model. Table 3a illustrates the OR, 95% CI, and *p*-values from the multilevel mixed-effects logistic regression model, which examines the association between HGS and the odds of drawing ability, word recall ability, and TICS-10 performance. It also reports the difference in means (“Mean Diff.”), 95% CI of the difference, and *p*-values from the multilevel linear mixed-effects model for the association between HGS and OCS.

In the unadjusted model, high HGS is associated with 230% higher odds of drawing ability (OR = 3.30; 95% CI: 2.93 to 3.71; *p* < 0.001), 208% higher odds of word recall ability (OR = 3.08; 95% CI: 2.27 to 4.19; *p* < 0.001), and 280% higher odds of TICS-10 performance (OR = 3.80; 95% CI: 3.30 to 4.37; *p* < 0.001). For the continuous OCS, subjects with high HGS, on average, have a 1.36 (difference in mean = 1.36; 95% CI: 1.21 to 1.52; *p* < 0.001) higher mean of the overall cognition score than those with low HGS.

For the multivariable model adjusting for follow-up time, baseline age, gender, education level, BMI, and household income, high HGS is associated with 34% higher odds of drawing ability (OR = 1.34; 95% CI: 1.21 to1.60; *p* = 0.001) and 37% higher odds of high TICS-10 scores (OR = 1.37; 95% CI: 1.11 to 1.69; *p* = 0.003). For OCS, subjects with high HGS, on average, have a 0.50 (difference in mean = 1.36; 95% CI: 0.27 to 0.72; *p* < 0.001) higher mean of overall cognition score than those with low HGS.

Similar to Table 2a, Table 3b reports the estimates, 95% CIs, and *p*-values for the association between cognitive outcomes and lagged HGS scores (and current HGS scores as an adjusting variable). The lagged HGS scores are statistically significantly associated with all cognitive outcomes in the model that only adjusts for the current GS and follow-up time (Model A). In the multivariable model adjusting for current HGS, follow-up time, baseline age, gender, education level, BMI, and household income (Model B), the lagged HGS is not significantly associated with any of the cognitive functions. However, in Model B, current HGS status is significantly associated with all cognitive functions except for word recall.

### 3.4. Impact of Baseline GS on Temporal Change in Cognitive Functions

We examined the influence of baseline GS (high vs. low) on temporal changes in OCS among older adults in CHARLS over the three study waves. Table 4 reports the slope for both low-GS and high-GS groups and the difference in slopes between the two groups. Analyses were performed for the entire study cohort, for males only, and for females only.

Notably, GS significantly modifies the slope (temporal trend) of OCS from Waves 1 to 3 in both the unadjusted and adjusted models. In the adjusted model, the OCS for subjects with high GS at baseline is expected to decline by 0.19 per year (95% CI: −0.25 to −0.13), while the cognitive score for subjects with low GS over the three waves is expected to decline by 0.35 per year (95% CI: −0.43 to −0.28). The slope of OCS decline for individuals with high GS is significantly less than for those with low GS (diff. in slope = 0.16; 95% CI: 0.06 to 0.27; *p*-value < 0.001).

Gender-specific analysis identified that the temporal trend of OCS was significantly modified by GS among male older adults in the adjusted model (diff. in slope = 0.19, 95% CI: 0.04 to 0.35, *p*-value = 0.015). Males with low baseline GS had a 0.36/year decline in OCS (slope = −0.36; 95% CI: −0.49 to −0.24) over the three waves. Although the males with high baseline GS still have an OCS decline (slope = −0.17; 95% CI: −0.25 to −0.08), their decline is much less severe. On the other hand, the temporal trend of OCS was not modified by GS among females in the adjusted model (diff. in slope = 0.13; 95% CI: −0.01 to 0.27; *p*-value = 0.067) and the unadjusted model (diff. in slope = 0.13; 95% CI: −0.01 to 0.27; *p*-value = 0.062). This implies that GS would be more important in predicting future cognitive functions for older males than for older females using primary care.

### 3.5. Impact of Baseline HGS on Temporal Changes in Cognitive Functions

We also examined the impact of baseline HGS (high vs. low) on temporal changes in OCS over three waves among middle-aged and older adults in CHARLS. Table 5 reports the slope for both low-HGS and high-HGS groups, and the difference in slopes between the two groups. Analysis was performed for the entire study cohort, only for males, and only for females.

HGS significantly modifies the temporal trend (slope) of OCS from Wave 1 to 3 in both the unadjusted and adjusted models. In the adjusted model, the OCS for subjects with low HGS at baseline is expected to decline by 0.13 per year (95% CI: −0.17 to −0.09), while the cognitive score for subjects with high HGS is almost unchanged over the three waves (slope = −0.01; 95% CI: −0.06 to 0.03). This ends up with a statistically significant difference in the slope of OCS between high and low baseline HGS (diff. in slope = 0.12; 95% CI: 0.05 to 0.18; *p*-value < 0.001).

Gender-specific analysis identified that the temporal trend of OCS was significantly modified by HGS for females in the adjusted model (diff. in slope = 0.23; 95% CI: 0.11 to 0.34; *p*-value < 0.001). Females with low baseline HGS have a significant decline (slope = −0.16; 95% CI: −0.21 to −0.11) in OCS, while the OCS for females with high HGS did not significantly change over the three waves (slope = 0.07, 95% CI: −0.03 to 0.16). However, the temporal trend of OCS was not modified by HGS for males in the adjusted model (diff. in slope = 0.11, 95% CI: −0.01 to 0.24, *p*-value = 0.059). This implies that HGS would be more important to predict future cognitive decline for middle-aged and older females using primary care than for their male counterparts.

## 4. Discussion

In this research, we leveraged a longitudinal study design and performed a longitudinal data analysis using Wave 1–3 data from CHARLS. To the best of our knowledge, this is the first comprehensive longitudinal analysis to examine the relationship between cognitive functions and physical functions (GS and HGS) in middle-aged and older adults in primary health care.

Restricted by the data availability in CHARLS, we could only investigate associations between GS and cognitive functions among older adults (60 years of age and older), since only older adults underwent the walking gait tests in CHARLS. Thus, the findings regarding GS and cognitive functions are more relevant for older adults. HGS was measured for all subjects in CHARLS (adults 45 years of age and above), which allowed us to perform the analysis for all eligible subjects and subjects 60 years of age and above. To examine whether our conclusions can apply only to older adults, we performed a sub-analysis using only individuals aged 60 and above. All results regarding HGS in Section 3 are very similar between the primary study cohort (aged 45 and above) and the sub-cohort (aged 60 and above). All *p*-values in the sub-analysis were consistent with the significance in the primary analysis. So, the same conclusions regarding HGS and cognitive functions apply to the population of older adults.

Our analytical results demonstrate a significant association between cognitive functions and their concurrent physical functions (HGS and GS) among middle-aged and older adults using primary care. This is consistent with previous studies based on cross-sectional data [11,12,14,26] and longitudinal data [13,25]. The cross-sectional association between cognitive functions and GS/HGS has been well reported. In a systematic review by Clouston et al. [44], the authors summarized multiple studies among older adults to assess the relationships between physical and cognitive functions. Their research showed that both stronger HGS and faster GS were associated with better cognitive scores. Our study confirmed this cross-sectional relationship among middle-aged and older adults in China.

In this study, we did not find that HGS at a lagged time (one wave lag) was associated with any of the four cognitive functions in the adjusted model. This result coincides with Clouston’s research [44], which reported that only GS consistently predicted cognitive functions, while HGS showed less consistent associations over time. However, the result is not in agreement with a previous longitudinal study using data from the Korean Longitudinal Study of Aging (KLoSA) [45], in which the authors examined the bidirectional relationship between HGS and cognitive impairment in older adults over 8 years. The study used a time-lagged (lagged by two waves) analysis, in which the exposure occurred 2 years before the outcome, and found that HGS at the lagged time was associated with a subsequent reduction in the risk of cognitive impairment, while cognitive impairment predicted a decline in HGS. On the other hand, GS at a lagged time is significantly associated with all four cognitive functions in the adjusted model. This finding agrees with another longitudinal study using CHARLS data [46], which reported that current cognitive function (e.g., global cognition and mental intactness) was significantly associated with subsequent GS, and vice versa.

Our study finds a significant difference in the subsequent decline in OCS between different baseline HGS and GS status (high versus low) in middle-aged and older adults using primary care. Subjects with high HGS and high GS at baseline did not show a severe decline in OCS within 4–5 years from baseline, while a significant decline in OCS was found among middle-aged and older adults with low HGS and low GS. This is consistent with the conclusions of previous studies [7,47,48,49]. For example, Rosso et al. [47] investigated the relationship between GS and cognitive decline in older adults and found that slower GS was associated with an increased risk of cognitive decline. Hackett et al. [48] conducted a longitudinal study using data from the English Longitudinal Study of Ageing (ELSA). They argued that individuals with faster baseline GS had a lower risk of developing cognitive dysfunction. Chai et al. [7] also used the CHARLS data to conduct a longitudinal analysis over a 3-year follow-up period and found that higher HGS was associated with better subsequent cognitive functions. However, the study conducted by Hooghiemstra [50] suggested that HGS and GS are related to current cognitive status and modestly related to cognitive decline. However, based on the time-to-event analysis with the Cox proportional hazards model, the authors found that HGS and GS are not useful as early markers of incident clinical progression as they are not significantly associated with the hazard of mild cognitive impairment (MCI) events. This may imply that GS and HGS are better physical performance markers for longitudinal cognitive decline than for the prognosis of cognitive impairment events. We could not verify this hypothesis in this study since time-to-event data for MCI (or other cognitive impairments) are not available in CHARLS.

Notably, the significant association between GS/HGS and cognitive function may not exist in certain sub-populations. Another study using the KLoSA data [51] found no significant association between HGS and cognitive impairment in non-obese older women. This implies that gender and body mass index could modify the relationship between HGS and cognitive function in this population. Future studies are needed to explore more potential moderators for the association between GS/HGS and cognitive functions.

Our study also finds that baseline GS is predictive of subsequent changes in cognitive scores only for men, and baseline HGS is predictive of subsequent changes in cognitive scores only for women. Our hypothesis is that lower body strength could be more important for neuromuscular functions in men, as men normally engage in heavier physical activities (whether work-related or not) which require for stronger lower body strength for neuromuscular efficiency. A better GS reflects better lower body strength and would therefore be related to better cognitive functioning in men. In contrast, HGS primarily reflects upper body strength and fine motor coordination, which are often better-preserved in women and may play a more significant role in their cognitive functioning. Future studies are warranted to further investigate these gender-specific associations and examine our hypothesis.

As for the association between HGS and subsequent cognitive decline, a recent longitudinal study by Feng and colleagues [52] explored the association between HGS and MCI among individuals residing in rural areas of China. The authors sought to assess the association of HGS with cognitive impairment and the risk of future incidents of MCI for both males and females. The study found that although women in the lowest quintile of HGS had an increased risk of incident MCI compared with women in the highest quintile of HGS, a similar association was not found among men. Our findings agree with Feng et. al., even if we only focus on the temporal change in the cognitive score instead of the incidence of MCI. Another study by Lee et al. [53], using data from the Korean Longitudinal Study of Aging (2006–2018), reported that males in the lowest quartile of baseline HGS experienced significantly decreased cognitive function over time compared to males in the highest quartile, but females in both the lowest and the second lowest quartiles experienced significantly decreased cognitive function. Combining our study findings (based on dichotomizing HGS by the upper and lower halves) and the results from Lee et al. suggests that baseline HGS is more sensitive to cognitive function decline among older females in East Asia. Among older males in East Asia, only those who are very weak in HGS may be at risk of significant cognitive decline. A recent European study conducted by Haagsma and colleagues [54] investigated the bidirectional associations between HGS and multiple cognitive functions (including memory, verbal fluency, word recall, and numeracy) among European older adults. The researchers found that HGS predicted cognitive decline in both sexes, except for in numeracy in men. The strongest association with HGS in women was with verbal fluency, and the strongest association with HGS in men was with word recall. The results from Haagsma’s study are not in agreement with our findings in the sex-stratified analysis. A secondary analysis in our study did not find a significant association between HGS and word recall in men. However, readers should note that the population of Haagsma’s study is different from our study population. As for the impact of GS on subsequent cognitive decline, our results agree with many previous studies and disagree with several others. For example, a study conducted by Bun et al. [55] found that female and male Japanese participants in the lowest GS quintile had a significantly increased risk of MCI compared to the fastest GS quintile. In sex-specific analyses, only males showed associations between GS and MCI. However, Best and colleagues [48] reported that the association between GS and cognitive decline was similar for women and men.

The primary goal of this work is to evaluate the association between GS/HGS (as exposures) and cognitive functions using longitudinal data analyses. Although the assessment of the causal relationship between GS/HGS and cognitive functions is not a primary goal of this work, we posit that it is more likely that improvements in GS/HGS may lead to better cognitive functions than the reverse. To that end, Bradford Hill criteria [56] were used to examine the potential reverse causality. Although we found that the previous cognitive functions were significantly (all *p*-values > 0.03 but <0.05), but not strongly, associated with both HGS and GS measured subsequently, most items in the Bradford Hill criteria were not satisfied in this study. Therefore, we are inclined to claim that cognitive functions are more likely to be the outcomes of GS/HGS. Future works are warranted to investigate more causal relationships between GS/HGS and cognitive functions among middle-aged and older adults.

Health interventions involving physical exercise have been reported to improve cognitive functions among older adults in the literature. In a narrative review, Quigley and colleagues [57] summarized the effects of chronic physical activity (PA) and exercise on cognitive functions and the main biological mechanisms underlying exercise-induced cognitive improvements. These mechanisms include the upregulation of growth factors and neuroplasticity, inhibition of inflammatory biomarker production, improved vascular function, and hypothalamic–pituitary–adrenal axis regulation. In a recent meta-analysis, Valenzuela et al. [58] found that physical exercises could significantly improve almost all physical functions in older adults (aged from 67 years to 92 years old), including GS and HGS. Several works reported that interventions integrating a PA component with cognitive tasks improved both PA and cognitive functions among older adults. For example, a randomized trial by Abo and Hamaguchi [59] found that engaging in the dual-task exercises intervention (combining physical exercise with cognitive tasks) effectively enhanced both physical and cognitive functions in older adults. Another study, conducted in Singapore, showed that their multicomponent exercise program improved physical functions and cognition among pre-frail older adults ≥ 65 years attending primary care clinics [60]. The intervention led to a significant improvement in cognition and amelioration in depression, physical function, muscle mass, frailty, perceived health, and TNF-α levels.

In a secondary analysis of this study, we also found that individuals’ baseline HGS is significantly associated with all types (light, moderate, and vigorous) of PA, while GS is only associated with moderate PA. Thus, we hypothesize that PA interventions may potentially improve cognitive functions through enhancement in HGS and GS among older adults, and in moderate PA it is more important to target the improvement of GS. Future studies are warranted to explore whether PA interventions targeting GS and HGS among older adults with slow GS or weak HGS can prevent them from cognitive decline.

Although our study focuses on middle-aged and older adults who participated in CHARLS and used primary health care, most of our conclusions are consistent with previous studies based on the general population. For example, a prior study [7] using a general CHARLS population (not restricted to primary health care users, but with a shorter follow-up period) also found that higher HGS was associated with better subsequent cognitive functions. Our findings would be particularly valuable for primary care providers (PCPs) and primary care policy makers for decision-making and patient education.

Like most public health research, our study suffers from some limitations. First, the calculation of GS was based on the time of 2.5 m walking along a 4 m, non-carpeted walking course. There were two measurements of walking time for each participant. We used the shorter time of the two to calculate GS, instead of the average time, as a large proportion of the subjects only had one measurement. In addition, since most of the CHARLS surveys were conducted at survey participants’ homes, there could be a large person-to-person variation in the preparation of the 4 m, non-carpeted walking course and in personal preferences in walking short distances. For the above reasons, the GS measurements and calculations may be subject to bias. Second, many variables considered in this study (such as age, education, and household income) are self-reported, so these measurements are subject to bias. Third, this study is a secondary data analysis of CHARLS, so all study subjects are Chinese. Although this study has a relatively large sample size, our findings may be more relevant to East Asian populations of middle-aged and older adults using primary care. It may not be generalizable to populations of other races/ethnicities, geographical areas, or other clinical settings. Future studies based on data from different populations or clinical settings are needed to examine whether our findings can be reproduced in those populations and clinical settings. Finally, in our analyses, we dichotomized original measurements of GS and HGS to improve interpretability, address potential model convergence issues, and ensure consistency across analyses. However, we acknowledge that this approach may lead to a loss of information.

## 5. Conclusions

This study comprehensively examines the longitudinal relationships between cognitive functions and HGS and between cognitive functions and GS among middle-aged and older adults in primary care in China. Our findings show that both GS and HGS are positively associated with concurrent cognitive functions. GS at the lagged time is positively associated with all four cognitive functions, while HGS at a lagged time is not associated with any of the four cognitive functions. The study also identifies a significant difference in the subsequent decline in OCS between older adults with different HGS and GS status (high versus low) at baseline. In the gender-specific analysis, GS is found to be predictive of the subsequent change in cognitive scores among males, while HGS is predictive of the subsequent change in cognitive scores among females.

Considering our study findings, we propose the following recommendations to researchers and healthcare professionals. First, developing health interventions specifically tailored to managing muscle strength and walking gait may help middle-aged and older adults prevent cognitive decline and reduce the personal and public load on primary healthcare for aging countries. These interventions could include community-based group health programs or telehealth physical education. Second, among older adults in Asia (especially East Asia), early intervention in cognitive impairment can be determined based on the individuals’ GS and HGS, especially in primary health care. GS is more important for older male adults, and HGS is more important for older female adults. Finally, future research is needed to investigate the longitudinal relationships between cognitive functions and GS/HGS among more diverse populations and clinical settings.

## Figures and Tables

**Table 1:** (**a**): Baseline characteristics of the GS cohort (N = 1903). (**b**): Baseline characteristics of the HGS cohort (N = 4218).

(**a**)								
**Variables**		**All Subjects** **(N = 1903)**		**Baseline GS Status**				
				**Low GS** **(N = 964, 50.66%)**		**High GS** **(N = 939, 49.34%)**		***p*** ^1^
		**N (%)**	**Mean (SD)**	**N (%)**	**Mean (SD)**	**N (%)**	**Mean (SD)**	
**Age at baseline (years)**			67.43 (5.99)		68.80 (6.47)		66.03 (5.09)	<0.001
**Gender**	Male	880 (46.24)		377 (39.11)		503 (53.57)		<0.001
	Female	1023 (53.76)		587 (60.89)		436 (46.43)		
**Education Level**	Less than lower secondary	1842 (96.79)		949 (98.44)		893 (95.10)		<0.001
	Upper secondary and vocational training or above	61 (3.21)		15 (1.56)		46 (4.90)		
**BMI**	Underweight	218 (11.46)		118 (12.24)		100 (10.65)		0.499
	Normal weight	853 (44.82)		427 (44.29)		426 (45.37)		
	Overweight	355 (18.65)		173 (17.95)		182 (19.38)		
	Obesity	457 (24.01)		23 (24.17)		224 (23.86)		
	Missing	20 (1.05)		13 (1.35)		7 (0.75)		
**Household Income**	Low	727 (38.20)		408 (42.32)		319 (33.97)		<0.001
	High	726 (38.15)		342 (35.48)		384 (40.89)		
	Missing	450 (23.65)		214 (22.20)		236 (25.13)		
**Able to Draw Assigned Picture**	No	912 (47.92)		520 (53.94)		392 (41.75)		<0.001
	Yes	928 (48.77)		400 (41.49)		528 (56.23)		
	Missing	63 (3.31)		44 (4.56)		19 (2.02)		
**TICS-10**	Low	915 (48.08)		536 (55.60)		379 (40.36)		<0.001
	High	904 (47.50)		370 (38.38)		534 (56.87)		
	Missing	84 (4.41)		58 (6.02)		26 (2.77)		
**Word Recall**	Low	311 (16.34)		195 (20.23)		116 (12.35)		<0.001
	High	1508 (79.24)		711 (73.76)		797 (84.88)		
	Missing	84 (4.41)		58 (6.02)		26 (2.77)		
**Overall Cognition Score (*N* = 1859)**			10.02 (4.55)		8.96 (4.54)		11.09 (4.30)	<0.001
(**b**)								
**Variables**		**All Subjects** **(N = 4218)**		**By Baseline HGS Status**				
				**Low HGS** **(N = 2186, 51.83%)**		**High HGS** **(N = 2032, 48.17%)**		***p*** ^1^
		**N (%)**	**Mean (SD)**	**N (%)**	**Mean (SD)**	**N (%)**	**Mean (SD)**	
**Age at Baseline (years)**			59.38(9.22)		61.23 (9.55)		57.40 (8.42)	<0.001
**Gender**	Male	1756 (41.63)		325 (14.87)		1431 (70.42)		<0.001
	Female	2462 (58.37)		1861(85.13)		601 (29.58)		
**Education Level**	Less than lower secondary	3928 (93.12)		2109 (96.48)		1819 (89.52)		<0.001
	Upper secondary and vocational training or above	290 (6.88)		77 (3.52)		213 (10.48)		
**BMI**	Underweight	337 (7.99)		224 (10.25)		113 (5.56)		<0.001
	Normal weight	1710 (40.54)		861 (39.39)		849 (41.78)		
	Overweight	817 (19.37)		396 (18.12)		421 (20.72)		
	Obesity	1301 (30.84)		673 (30.79)		628 (30.91)		
	Missing	53 (1.26)		32 (1.46)		21 (1.03)		
**Household Income**	Low	1383 (32.79)		780 (35.68)		603 (29.68)		<0.001
	High	1382 (32.76)		705 (32.25)		677 (33.32)		
	Missing	1453 (34.45)		701 (32.07)		752 (37.01)		
**Able to Draw Assigned Picture**	No	1724 (40.87)		1133 (51.83)		591 (29.08)		<0.001
	Yes	2386 (56.57)		981 (44.88)		1405 (69.14)		
	Missing	108 (2.56)		72 (3.29)		36 (1.77)		
**TICS-10**	Low	2252 (53.39)		1388 (63.49)		864 (42.52)		<0.001
	High	1833 (43.46)		703 (32.16)		1130 (55.61)		
	Missing	133 (3.15)		95 (4.35)		38 (1.87)		
**Word Recall**	Low	512 (12.14)		394 (18.02)		118 (5.81)		<0.001
	High	3573 (84.71)		1697 (77.63)		1876 (92.32)		
	Missing	133 (3.15)		95 (4.35)		38 (1.87)		
**Overall Cognition Score** **(N = 4147)**			10.96(4.39)		9.71 (4.49)		12.28 (3.88)	<0.001

^1^ *p*-values based on two-sample *t* tests for continuous variables and Chi-squared tests for categorical variables.

**Table 2:** (**a**): The association between cognitive functions and concurrent GS. (**b**): The association between cognitive functions and GS at a lagged time (one wave lag) and current time.

(**a**)					
**Outcome**	**Statistics**	**Unadjusted Model**		**Adjusted Model ^1^**	
**Drawing Ability**	OR	1.89		1.33	
	95% CI	(1.58, 2.27)		(1.07, 1.66)	
	*p*-value	<0.001		0.010	
**Word Recall Ability**	OR	1.67		2.19	
	95% CI	(1.17, 2.40)		(1.14, 4.18)	
	*p*-value	0.005		0.018	
**TICS-10**	OR	2.31		1.52	
	95% CI	(1.85, 2.89)		(1.15, 2.00)	
	*p*-value	<0.001		0.003	
**Overall Cognition**	Mean Diff.	0.78		0.57	
	95% CI	(0.57, 0.99)		(0.32, 0.82)	
	*p*-value	<0.001		<0.001	
(**b**)					
**Outcome**	**Statistics**	**Adjusted Model A** ^2^		**Adjusted Model B** ^3^	
		**Lagged GS**	**Current GS**	**Lagged GS**	**Current GS**
**Drawing Ability**	OR	1.69	2.04	1.21	1.31
	95% CI	1.37, 2.09	1.63, 2.55	0.92, 1.60	0.99, 1.73
	*p*-value	<0.001	<0.001	0.162	0.063
**Word Recall Ability**	OR	2.76	2.53	1.48	3.60
	95% CI	1.48, 5.06	1.35, 4.76	0.50, 4.34	1.17, 11.06
	*p*-value	0.001	0.004	0.477	0.025
**TICS-10**	OR	2.52	2.62	1.36	1.48
	95% CI	1.90, 3.34	2.05, 3.70	0.95, 1.94	1.02, 2.16
	*p*-value	<0.001	<0.001	0.089	0.039
**Overall Cognition**	Mean Diff.	1.40	1.30	0.73	0.79
	95% CI	1.11, 1.69	1.02, 1.58	0.37, 1.10)	0.44, 1.14
	*p*-value	<0.001	<0.001	<0.001	<0.001

^1^ Model adjusted for follow-up time, baseline age, gender, education level, BMI, and household income. Education level was dichotomized to at least upper secondary education (including individuals having upper secondary, vocational, or tertiary education) and lower than upper secondary education. ^2^ Model adjusted for current GS, and follow-up time. ^3^ Model adjusted for current GS, follow-up time, baseline age, gender, education level, BMI, and household income. Education level was dichotomized to at least upper secondary education (including individuals having upper secondary, vocational, or tertiary education) and lower than upper secondary education.

**Table 3:** (**a**): The association between cognitive functions and concurrent HGS. (**b**): The association between cognitive functions and HGS at a lagged time (one wave lag) and current time.

(**a**)					
**Outcome**	**Statistics**	**Unadjusted Model**		**Adjusted Model ^1^**	
**Drawing Ability**	OR	3.30		1.34	
	95% CI	2.93, 3.71		1.12, 1.60	
	*p*-value	<0.001		0.001	
**Word Recall Ability**	OR	3.08		1.42	
	95% CI	2.27, 4.19		0.78, 2.57	
	*p*-value	<0.001		0.249	
**TICS-10**	OR	3.80		1.37	
	95% CI	3.30, 4.37		1.11, 1.69	
	*p*-value	<0.001		0.003	
**Overall Cognition**	Mean Diff.	1.36		0.50	
	95% CI	1.21, 1.52		0.27, 0.72	
	*p*-value	<0.001		<0.001	
(**b**)					
**Outcome**	**Statistics**	**Adjusted Model A** ^2^		**Adjusted Model B** ^3^	
		**Lagged HGS**	**Current HGS**	**Lagged HGS**	**Current HGS**
**Drawing Ability**	OR	2.09	2.46	1.13	1.38
	95% CI	1.79, 2.43	2.10, 2.89	0.90, 1.41	1.10, 1.74
	*p*-value	<0.001	<0.001	0.306	0.005
**Word Recall**	OR	2.19	2.61	2.15	1.31
**Ability**	95% CI	1.31, 3.66	1.48, 4.61	0.52, 9.00	0.31, 5.46
	*p*-value	0.003	0.001	0.293	0.715
**TICS-10**	OR	2.25	3.34	1.19	1.42
	95% CI	1.85, 2.72	2.76, 4.04	0.91, 1.55	1.09, 1.86
	*p*-value	<0.001	<0.001	0.212	0.010
**Overall Cognition**	Mean Diff.	1.26	1.60	0.18	0.54
	95% CI	1.07, 1.46	1.49, 1.89	−0.12, 0.49	0.24, 0.85
	*p*-value	<0.001	<0.001	0.242	<0.001

^1^ Model adjusted for follow-up time, baseline age, gender, education level, BMI, and household income. Education level was dichotomized to at least upper secondary education (including individuals having upper secondary, vocational, or tertiary education) and lower than upper secondary education. ^2^ Model adjusted for current HGS, and follow-up time. ^3^ Model adjusted for current HGS, follow-up time, baseline age, gender, education level, BMI, and household income. Education level was dichotomized to at least upper secondary education (including individuals having upper secondary, vocational, or tertiary education) and lower than upper secondary education.

**Table 4:** Impact of baseline GS on the temporal trend (slope) of overall cognitive functions.

**Overall Cognitive Score** **(All Subjects)**	**Unadjusted Model**			
	Slope (95% CI): Low GS	Slope (95% CI): High GS	Diff. in slope (High – Low GS)	*p* ^1^
	−0.35 (−0.43, −0.28)	−0.18 (−0.25, −0.13)	0.17 (0.07, 0.27)	<0.001
	**Adjusted Model ^2^**			
	Slope (95% CI): Low GS	Slope (95% CI): High GS	Diff. in slope (High – Low GS)	*p* ^1^
	−0.35 (−0.43, −0.28)	−0.19 (−0.25, −0.13)	0.16 (0.06, 0.27)	<0.001
**Overall Cognitive Score** **(Males Only)**	**Unadjusted Model**			
	Slope (95% CI): Low GS	Slope (95% CI): High GS	Diff. in slope (High – Low GS)	*p* ^1^
	−0.35 (−0.48, −0.23)	−0.17 (−0.25, −0.08)	0.18 (0.03, 0.35)	0.018
	**Adjusted Model** ^2^			
	Slope (95% CI): Low GS	Slope (95% CI): High GS	Diff. in slope (High – Low GS)	*p* ^1^
	−0.36 (−0.49, −0.24)	−0.17 (−0.25, −0.08)	0.19 (0.04, 0.35)	0.015
**Overall Cognitive Score** **(Females Only)**	**Unadjusted Model**			
	Slope (95% CI): Low GS	Slope (95% CI): High GS	Diff. in slope (High – Low GS)	*p* ^1^
	−0.34 (−0.44, −0.24)	−0.21 (−0.30, −0.12)	0.13 (−0.01, 0.27)	0.062
	**Adjusted Model** ^2^			
	Slope (95% CI): Low GS	Slope (95% CI): High GS	Diff. in slope (High – Low GS)	*p* ^1^
	−0.35 (−0.44, −0.25)	−0.22 (−0.31, −0.13)	0.13 (−0.01, 0.27)	0.067

^1^ *p*-value based on a *t*-test for the difference in slopes between high and low GS. ^2^ Model adjusted for baseline age, gender, education level, BMI, and household income. Education level is dichotomized to at least upper secondary education (including individuals having upper secondary, vocational, or tertiary education) and lower than upper secondary education.

**Table 5:** Impact of baseline HGS on the temporal trend (slope) of overall cognitive functions.

**Overall Cognitive Score** **(All Subjects)**	**Unadjusted Model**			
	Slope (95% CI): Low HGS	Slope (95% CI): High HGS	Diff. in slope (High – Low HGS)	*p* ^1^
	−0.13 (−0.17, −0.09)	−0.01 (−0.06, 0.03)	0.12 (0.05, 0.18)	<0.001
	**Adjusted Model ^2^**			
	Slope (95% CI): Low HGS	Slope (95% CI): High HGS	Diff. in slope (High – Low HGS)	*p* ^1^
	−0.16 (−0.20, −0.12)	−0.05 (−0.10, −0.01)	0.11 (0.04, 0.17)	<0.001
**Overall Cognitive Score** **(Males Only)**	**Unadjusted Model**			
	Slope (95% CI): Low HGS	Slope (95% CI): High HGS	Diff. in slope (High – Low HGS)	*p* ^1^
	−0.18 (−0.29, −0.08)	−0.06 (−0.11, −0.01)	0.13 (0.01, 0.25)	0.039
	**Adjusted Model** ^2^			
	Slope (95% CI): Low HGS	Slope (95% CI): High HGS	Diff. in slope (High – Low HGS)	*p* ^1^
	−0.20 (−0.31, −0.10)	−0.09 (−0.14, 0.03)	0.11 (−0.01, 0.24)	0.059
**Overall Cognitive Score** **(Females Only)**	**Unadjusted Model**			
	Slope (95% CI): Low HGS	Slope (95% CI): High HGS	Diff. in slope (High – Low HGS)	*p* ^1^
	−0.13 (−0.18, −0.09)	0.09 (−0.01, 0.19)	0.22 (0.11, 0.34)	<0.001
	**Adjusted Model** ^2^			
	Slope (95% CI): Low HGS	Slope (95% CI): High HGS	Diff. in slope (High – Low HGS)	*p* ^1^
	−0.16 (−0.21, −0.11)	0.07 (−0.03, 0.16)	0.23 (0.11, 0.34)	<0.001

^1^ *p*-value based on a *t*-test for the difference in slopes between high and low GS. ^2^ Model adjusted for baseline age, gender, education level, BMI, and household income. Education level was dichotomized to at least upper secondary education (including individuals having upper secondary, vocational, or tertiary education) and lower than upper secondary education.

## Data Availability

The data used in this study were obtained from the publicly available China Health and Retirement Longitudinal Study (CHARLS) database, which is hosted by the National School of Development at Peking University. The CHARLS dataset is accessible to researchers through an application process to ensure compliance with privacy and ethical considerations. Researchers can request access to the data at http://charls.pku.edu.cn/en (accessed on 29 April 2025).

## Abbreviations

The following abbreviations are used in this manuscript:

CHARLS	China Health and Retirement Longitudinal Study
CI	Confidence interval
HGS	Hand grip strength
GS	Gait speed
GLMM	Generalized linear mixed-effects model
OCS	Overall cognitive score
OR	Odds ratio
PA	Physical activity
